# Population Carrier Rates of Pathogenic *ARSA* Gene Mutations: Is Metachromatic Leukodystrophy Underdiagnosed?

**DOI:** 10.1371/journal.pone.0020218

**Published:** 2011-06-10

**Authors:** Agnieszka Ługowska, Joanna Ponińska, Paweł Krajewski, Grażyna Broda, Rafał Płoski

**Affiliations:** 1 Department of Genetics, Institute of Psychiatry and Neurology, Warsaw, Poland; 2 Department of Medical Genetics, Warsaw Medical University, Warsaw, Poland; 3 Department of Forensic Medicine, Warsaw Medical University, Warsaw, Poland; 4 Department of Cardiovascular Epidemiology and Prevention, and Health Promotion, Institute of Cardiology, Warsaw, Poland; Newcastle University, United Kingdom

## Abstract

**Background:**

Metachromatic leukodystrophy (MLD) is a severe neurometabolic disease caused mainly by deficiency of arylsulfatase A encoded by the *ARSA* gene. Based on epidemiological surveys the incidence of MLD per 100 000 live births varied from 0.6 to 2.5. Our purpose was to estimate the birth prevalence of MLD in Poland by determining population frequency of the common pathogenic *ARSA* gene mutations and to compare this estimate with epidemiological data.

**Methodology:**

We studied two independently ascertained cohorts from the Polish background population (N∼3000 each) and determined carrier rates of common *ARSA* gene mutations: c.459+1G>A, p.P426L, p.I179S (cohort 1) and c.459+1G>A, p.I179S (cohort 2).

**Principal Findings:**

Taking into account *ARSA* gene mutation distribution among 60 Polish patients, the expected MLD birth prevalence in the general population (assuming no selection against homozygous fetuses) was estimated as 4.0/100 000 and 4.1/100 000, respectively for the 1^st^ and the 2^nd^ cohort with a pooled estimate of 4.1/100 000 (CI: 1.8–9.4) which was higher than the estimate of 0.38 per 100 000 live births based on diagnosed cases. The p.I179S mutation was relatively more prevalent among controls than patients (OR = 3.6, P = 0.0082, for a comparison of p.I179S frequency relative to c.459+1G>A between controls vs. patients).

**Conclusions/Significance:**

The observed discrepancy between the measured incidence of metachromatic leukodystrophy and the predicted carriage rates suggests that MLD is substantially underdiagnosed in the Polish population. The underdiagnosis rate may be particularly high among patients with p.I179S mutation whose disease is characterized mainly by psychotic symptoms.

## Introduction

Metachromatic leukodystrophy (MLD; OMIM 250100) is a severe demyelinating disease inherited in an autosomal recessive fashion. MLD is caused by deficient activity of lysosomal hydrolase arylsulfatase A (ARSA; EC 3.1.6.8) which leads to sulfatide storage. Apart from an MLD type caused by saposin B deficiency all clinical forms of MLD are due to mutations in the *ARSA* gene located on chromosome 22q13. The overall incidence of MLD varies from 1 in 40 000 to 1 in 170 000 in different populations [1] most likely reflecting different pathogenic mutation carriage rates as well as differences in the diagnosis rate. Based on this data it can be assumed that carrier frequency for MLD varies from ∼1∶100 to 1∶130 implying that among ∼3500 individuals from the general population 17–35 persons should be MLD carriers. Three MLD causing mutations in the *ARSA* gene – c.459+1G>A, p.P426L, and p.I179S are responsible together for approximately 50% of mutated alleles in MLD patients from Poland [2].

The aim of this study was to estimate the birth incidence of MLD in Poland by determining the population frequency of the common MLD causing mutations and compare this estimate with epidemiological data.

## Results

### Screening population cohort 1

During the initial screening performed among subjects undergoing paternity testing results were obtained for 3 320, 3 467, and 3 159 samples, respectively for mutations c.459+1G>A, p.P426L, and p.I179S. Five heterozygous carriers for c.459+1G>A, seven heterozygous carriers for p.P426L, and 11 heterozygous carriers for p.I179S were identified (Table 1) yielding a joint population allele frequency of 0.35% (CI: 0.23–0.52). By taking into account that among Polish MLD patients these three mutations were present in 66 out of 120 chromosomes (55%), the population frequency of all other pathogenic *ARSA* gene mutations could be estimated as 0.28% (CI: 0.18–0.45) and joint population frequency of all pathogenic mutations in *ARSA* gene as 0.63% (CI: 0.41–0.98, Table 1). Based on these values the mutation carriage rate per 10 000 was calculated as 125 (CI: 81–194, Table 1) whereas the expected prevalence of fetuses with two pathogenic *ARSA* gene mutations was estimated as 4.0 per 100 000 conceived fetuses (or 1 in 25 000) with CI from 1.7 to 9.6.

**Table 1:** Screening for the most common *ARSA* gene mutations in a Polish population.

	c.459+1G>A	p.P426L	p.I179S	All mutations including unknown
***Population cohort 1***				
Individuals tested	3 320	3 467	3 159	3 315*
Observed heterozygotes	5	7	11	-
Gene frequency % (CI)	0.08 (0.03–0.18)	0.1 (0.05–0.21)	0.17 (0.1–0.31)	0.63 (0.41–0.98)
Carrier frequency per 10 000 (CI)	15 (6–35)	20 (10–42)	35 (19–62)	125 (81–194)
Affected per 100 000:				4.0 (CI: 1.7–9.6)
***Population cohort 2***				
Individuals tested	3 019	-	2 937	2 978*
Observed heterozygotes	4	-	11	-
Gene frequency % (CI)	0.07 (0.03–0.17)	-	0.19 (0.1–0.34)	0.64 (0.37–1.11)
Carrier frequency per 10 000 (CI)	13 (5–34)	-	37 (21–67)	128 (74–220)
Affected per 100 000:				4.1 (CI: 1.4–12.4)
***Population cohort 1+2***				
Individuals tested	6 339	-	6 096	6 218*
Observed heterozygotes	9	-	22	-
Gene frequency % (CI)	0.07 (0.04–0.13)	-	0.18 (0.12–0.27)	0.64 (0.42–0.97)
Carrier frequency per 10 000 (CI)	14 (7–27)	-	36 (24–55)	126 (84–192)
Affected per 100 000:				4.1 (CI: 1.8–9.4)

*mean number of subjects tested for each mutation.

We noted a trend for difference in the relative distribution of the tested *ARSA* gene mutations between MLD patients and controls. Among the patients the c.459+1G>A mutation was the most prevalent accounting for 42.4% or 28/66 of the three tested variants whereas p.I179S had lower frequency (28.8% or 19/66 of the tested variants). Among controls this correlation was reversed: c.459+1G>A was the least prevalent (21.7% or 5/23) whereas p.I179S had the highest frequency (47.8% or 11/23). When frequency of the p.I179S mutation was expressed relative to frequency of the c.459+1G>A mutation a trend with borderline statistical significance of P = 0.051 [Odds Ratio (OR) = 3.24, CI: 0.99–10.51] was observed in a comparison between controls (11/5) vs. patients (19/28, Table 2). The relative frequency of the p.P426L mutation was similar among patients (28.9% or 19/66) and controls (30.4% or 7/23, Table 2).

**Table 2:** Distribution of the tested mutations among controls and MLD patients.

Group/ mutation	c.459+1G>An	p.P426Ln (OR [CI]); P	p.I179Sn (OR [CI]); P
Population 1; n_mean_ = 3 315	5	7 (2.06 [0.59–7.20]); 0.27	11 (3.24 [0.99–10.51]); 0.051
Population 2; n_mean_ = 2 978	4	-	11 (4.05 [1.16–14.0]); 0.026
Population 1+2; n_mean_ = 6 218	9	-	22 (3.60 [1.37–9.43]); 0.0082
Patients, Poland; n = 60	28^1^	19^2^	19^3^

1 9 females, 12 males; ^2^ 5 females, 8 males; ^3^ 9 females, 10 males.

OR (Odds Ratio) and P value calculated for the frequency of p.P426L or p.I179S among controls vs. patients. For each mutation its frequency was expressed relative to the frequency of c.459+1G>A. For example, in case of p.I179S in Population 1+2: OR = (22/9) / (19/28) = 3.60.

### Screening population cohort 2

In order to verify these findings and further explore the trend for the increased frequency of p.I179S relative to c.459+1G>A among controls vs. patients we analyzed prevalence of these two mutations in an independently ascertained population cohort from the WOBASZ study (population cohort 2). Results were obtained for 3 019 and 2 937 samples for c.459+1G>A and p.I179S, respectively. Four heterozygous carriers of c.459+1G>A and 11 heterozygous carriers of p.I179S were identified yielding the respective estimates of gene frequency of 0.07% (CI: 0.03–0.17) and 0.19% (CI: 0.1–0.34, Table 1). Since these two mutations account for 39.2% (47/120) of the mutated chromosomes among patients the population frequency of all pathogenic *ARSA* gene mutations could be estimated as 0.64% (CI: 0.37–1.11, Table 1) and their carriage rate as 128 per 10 000 (CI: 74–220, Table 1). Thus, the expected prevalence of fetuses conceived with two MLD causing mutations is 4.1 per 100 000 conceived fetuses (or 1 in 24 390) CI from 1.4 to 12.4.

Analysis of frequency of the p.I179S mutation relative to c.459+1G>A showed a statistically significant difference (OR = 4.05, CI: 1.16–14.0, P = 0.026) between population cohort 2 (11/4) and patients (19/28, Table 2).

### Analysis of pooled population cohorts

Since there were no statistically significant differences between mutation frequency estimates based on the two population cohorts (P>0.8 in both comparisons) we also analyzed the data after pooling. This analysis was based on 6 339 subjects tested for c.459+1G>A, nine of whom were positive and 6 096 subjects tested for p.I179S of whom 22 were positive yielding the estimates of gene frequencies equal to 0.07% (CI: 0.04–0.13) and 0.18% (CI: 0.12–0.27), and carrier frequencies per 10 000 equal to 14 (CI: 7–27) and 36 (CI: 24–55), respectively for c.459+1G>A and p.I179S (Table 1).

By taking into account distribution of *ARSA* gene mutations among patients the gene frequency of all pathogenic mutations was estimated as 0.64 with CI from 0.42 to 0.97 and carriage rate per 10 000 as 126 (CI: 84–192, Table 1). Using the pooled cohort the expected prevalence of fetuses with two pathogenic *ARSA* gene mutations was estimated as 4.1 per 100 000 conceived fetuses (or 1 in 24 390) with CI from 1.8 to 9.4 (Table 1).

Analysis of frequency of the p.I179S mutation relative to c.459+1G>A showed a statistically significant difference (OR = 3.60, CI: 1.37–9.43, P = 0.0082) between pooled population cohorts (22/9) and patients (19/28, Table 2).

### Prevalence of MLD in Poland based on diagnosed cases

**Figure 1:**
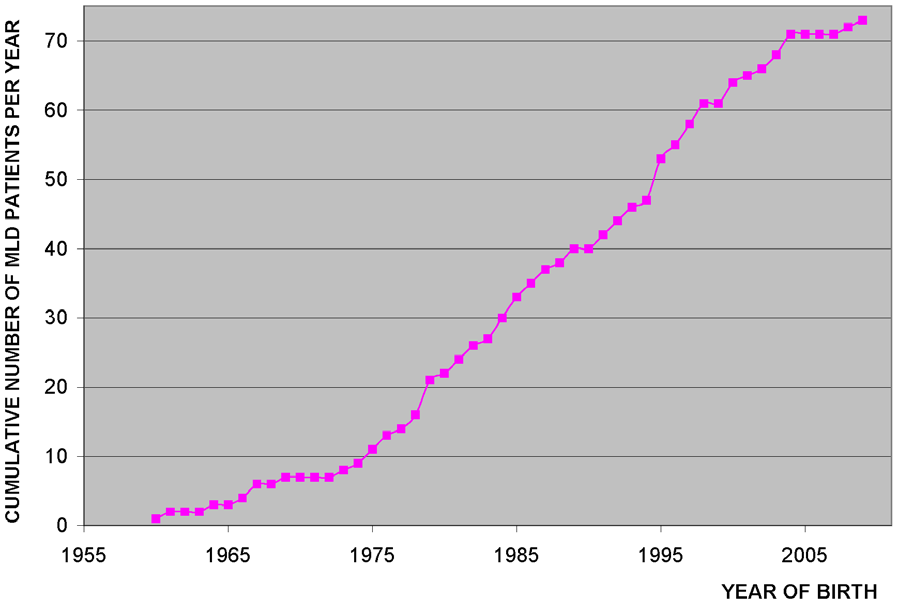
Cumulative number of new MLD cases diagnosed per year plotted against year of birth. The period from years 1975–2004 was used to calculate MLD incidence. The data used to draw the figure are shown in Table S1.

Analysis of the graph showing cumulative number of MLD cases diagnosed per year plotted against year of birth showed that the curve was linear from year 1975 to 2004 (Figure 1) indicating that this period was optimal for prevalence calculation [3]. By dividing total number of MLD patients born in this period by number of all children born alive in Poland in the respective time we obtained an estimate of MLD incidence equal to 0.38 per 100 000 (Table S1).

## Discussion

In the present study we determined the general population carriage rates of three pathogenic *ARSA* gene mutations: c.459+1G>A, p.P426L, and p.I179S. After taking into account the frequency of these mutations among MLD patients, the carriage rate of all pathogenic *ARSA* gene mutations was estimated as 125/10 000 or 1/80 whereas disease prevalence among conceived fetuses was 4.0 per 100 000. These estimates were closely confirmed by analysis of an independently ascertained population cohort (the carriage rate of 128/10 000 and disease prevalence equal to 4.1 per 100 000). An analysis using a pooled cohort of > 6 000 subjects yielded carriage rate per 10 000 equal to 126 or 1 in 79 and prevalence of fetuses conceived with two pathogenic MLD causing mutations of 4.1 per 100 000 (CI: 1.8–9.4). We also observed that the p.I179S mutation was relatively more frequent among controls than patients. In particular when p.I179S frequency was expressed relative to c.459+1G>A a statistically significant difference was observed between controls vs. patients (OR = 3.6, P = 0.0082).

The expected prevalence of fetuses conceived with two pathogenic *ARSA* mutations in Poland (4.1 per 100 000) is substantially higher than the birth prevalence of MLD based on diagnosed cases (0.38 per 100 000 live births as determined by us or 0.53 per 100 000 (1 in 189 000) as previously reported in Poland [4]). Our estimate is also higher than similar estimates made in other Caucasian populations: 1 in 40 000 (i.e. 2.5/100 000) in Northern Sweden [5], 1 in 130 000 (i.e. 0.77/100 000) in France [6], 1 in 170 000 (i.e. 0.59/100 000) in Germany [7], 1.85/100 000 in Portugal [8], 1.42/100 000 in the Netherlands [9], 1.43/ 100 000 in Turkey [10], and 1/40 000 (i.e. 2.5/100 000) in the Washington State [11]. Notably, except for Northern Sweden [5], the Washington State [11] and Portugal [8] the reported prevalence is lower than the lower bound of the CI of our estimate (i.e. 1.8/100 000), thus indicating that the observed differences are statistically significant.

The birth prevalences of MLD were higher only in small, geographically or ethnically isolated communities like in Habbanite Jews in Israel (1 in 75) [12], Arab inhabitants of Israel (1 in 8000) [13], Navajo Indians (1 in 6400) [14], and Eskimos (1 in 2500) [15].

This discrepancy between our estimate and observed incidence of MLD in Poland may have several causes. It should be emphasized that all birth prevalence values reported so far were based on direct diagnoses whereas our estimations are the first based on the mutation carrier rates in general population. It is possible that mutations in the *ARSA* gene resulting in the severe phenotype can lead to prenatal or perinatal loss of affected fetuses thus decreasing prevalence among live born children. Whereas in Poland prenatal screening for MLD has not been performed, prenatal diagnosis in families with MLD probands is available which is likely to decrease number of affected individuals born. We also cannot fully exclude that some patients are diagnosed abroad and thus not referred to the Institute of Psychiatry and Neurology in Warsaw. However, this should not be frequent since in the Polish health system referral to foreign centers occurs only in exceptional cases.

Our findings may also be influenced by a distinct phenotype associated with the p.I179S and possibly other yet unknown mutations. Since patients with the p.I179S mutation suffer initially mainly from psychotic manifestations it is possible, that some individuals from this group are misdiagnosed as affected with atypical schizophrenia [16], [17]. The diagnostic issues may be particularly pronounced in case of p.I179S homozygotes since no such MLD patients have been described so far. In this context it is particularly noteworthy that we observed statistically significant inverse relationship between relative frequencies of p.I179S and c.459+1G>A mutations among patients vs. controls, consistent with possibility of underdiagnosis of cases with the former mutation. The problem of misdiagnosed patients is also actual for the adult onset patients in whom the beginning stages of the disease may be mild and difficult to detect.

The close similarity of carriage rates of c.459+1G>A and p.I179S in a population coming mainly from Central Poland (the paternity testing sample, cohort 1) and population representative of the whole country (the WOBASZ sample, cohort 2) supports ethnic homogeneity of Polish population and strengthens our conclusions which rely also on data from patients coming from the whole Polish territory. Homogeneity of contemporary Polish population is consistent with studies on distribution of Y chromosome markers which are particularly sensitive to population substructure [18], [19].

A limitation of our study is caused by lack of appropriate molecular testing allowing to rigorously exclude related individuals from the population cohorts studied. Further, whereas our determination of background population carriage rates is apparently robust the data on mutation distribution among patients may not be so reliable as they come from a single group of limited size. Although our statistical approach takes this into account it would be interesting to verify our conclusions in another population and MLD patients groups.

In conclusion, MLD and other lysosomal disorders are considered to be orphan diseases but the expected incidence of fetuses conceived with two pathogenic *ARSA* mutations of 4.1 in 100 000 and heterozygote frequency of 1 in 79 negate this statement. One possible reason for the discrepancy between observed and expected prevalence of MLD may be related to disease underdiagnosis, especially among patients with p.I179S mutation.

## Materials and Methods

### Population samples

#### Population cohort 1

Three thousand five hundred DNA samples were obtained from anonymous DNA samples collection of Department of Forensic Medicine WUM which has been described previously [20], [21]. These samples come from couples who underwent genetic testing because of disputed paternity (alleged fathers and mothers of tested children, no duplicates or obviously related individuals, all Caucasians living mainly in Central Poland, male:female ratio ∼1, mean age 30.7 years, standard deviation 10.0, range from 15 to 77 years). Informed consents were not obtained for this cohort, because the samples and the obtained data were analyzed anonymously. This protocol has been approved by and obtained a positive opinion from the Bioethics Committee at the Institute of Psychiatry and Neurology in Warsaw, which specifically approved the waiver of consent.

#### Population cohort 2

This cohort consisted of three thousand one hundred subjects randomly selected from the participants of WOBASZ study [22], [23]. The WOBASZ study was a cross-sectional epidemiological study based on subjects randomly selected from Polish population register of permanent residents aged 20–74 years. The sampling scheme was stratified by sex, administration units and type of urbanization. Data were collected from January 2003 to December 2005. The response rate was 70%. The study was approved by The Medical Ethics Committee of National Institute of Cardiology in Warsaw and each participant gave a written consent for taking blood sample for DNA isolation and genetic analyses [22], [23].

### Patients

The distribution of the c.459+1G>A, p.P426L and p.I179S mutations in the *ARSA* gene among MLD patients was determined based on 60 unrelated probands who were all typed for these variants for diagnostic purposes as described previously [24]. This group included 43 individuals described earlier [24] as well as 17 additional patients (Polish Caucasians, male: female ratio ∼1, age 1.5–51 yr) . Since the Department of Genetics at the Institute of Psychiatry and Neurology is the only one centre diagnosing MLD in Poland these patients can be regarded as being randomly selected across the national population.

### Typing

In population cohort 1 the screening for the c.459+1G>A, p.P426L, and p.I179S mutations was performed by PCR-RFLP methods [2]. In population cohort 2 we used a TaqMan method based on *Assay on Demand* from *Life Technologies* and real time PCR apparatus ABI Prism 7500. In all TaqMan experiments at least three positive samples verified by PCR-RFLP were used as controls. For all mutations positive results were verified by re-analysis of samples from the original stock.

### Statistical analysis

Joint population frequency of tested mutations (*f_tested_pop._*) was estimated by summing individual frequencies. The population frequency of mutations which were not tested including those which are unknown (*f_n.tested_pop._*) was estimated from the prevalence of the tested mutations among MLD patients from our center (*f_tested_pat._*). Assuming similar penetrance for all MLD mutations their relative frequencies among patients and population controls should be the same. Thus, *f_n.tested_pop._*  =  *f_tested_pop._* (1- *f_tested_pat._*)/ *f_tested_pat._*. Similarly, population frequency of all pathogenic mutations *f_all_pop_*  =  *f_tested_pop_* / *f_tested_pat._* Based on data available from patients *f_tested_pat._* was estimated as 66/120 or 55% for Population cohort 1 and 47/120 or 39.2% for Population cohort 2 as well as the pooled cohorts. The expected population frequencies of (i) carriers of pathogenic mutations and (ii) of affected individuals (i.e. those with two pathogenic *ARSA* gene mutations) were estimated from Hardy-Weinberg law as (i) 2× *f_all_pop_* × (1-*f_all_pop_*) and (ii) *f_all_pop_* ^2^.

Ninety five percent confidence intervals (CI) for mutation frequencies were calculated according to Wilson [25] whereas CI for the ratio *f_tested_pop_* / *f_tested_pat._* was calculated according to Miettinen and Nurminen as ratio of proportions allowing to take into account the uncertainty associated with estimates of both *f_tested_pop_* and *f_tested_pat._* [26]. During analysis of population cohort 1 for calculation of CI for *f_tested_pop_* it was assumed that 3 315 samples were tested which represents a mean of numbers actually tested for each mutation (i.e. 3 320, 3 467, and 3 159). Analogous approach was used for Population cohort 2 and pooled cohort. Computations of CI were done using spreadsheet downloaded from (accessed 20.01.2007):http://www.cardiff.ac.uk/medicine/epidemiology_statistics/research/statistics/ODDSRATIOANDRR.xls. Frequency of the p.I179S mutation expressed relative to frequency of the c.459+1G>A in the general population and among patients was compared by a chi square test with one degree of freedom using *Statistica* software package.

### Incidence of MLD in Poland based on diagnosed cases

Incidence (birth prevalence) of MLD in Poland was determined by the method proposed by Claussen et al. [3]. This method is based on sorting all ascertained cases according to birth year and plotting the cumulative number of cases diagnosed per year against the year of birth. The linear region of the obtained curve indicates the period free from ascertainment errors due to inefficient registration (typically in the past) or yet undiagnosed cases in young subjects (most recent period). In order to estimate disease incidence, for the time period thus identified the total number of cases is divided by total number of births [3].

In Poland lysosomal disorders including MLD are diagnosed exclusively in the Department of Genetics at the Institute of Psychiatry and Neurology in Warsaw and it was assumed that all Polish patients with the suspicion of MLD were referred to our laboratory. The numbers of births per year were obtained from Polish Central Statistical Office (www.stat.gov.pl/gus).

## Supporting Information

Table S1 Demographic data vs. numbers of MLD patients born in Poland.
(DOC)
