# Supplementary material for: Population Carrier Rates of Pathogenic ARSA Gene Mutations: Is Metachromatic Leukodystrophy Underdiagnosed?
Source: PLoS One. 2011 Jun 10;6(6):e20218. doi: 10.1371/journal.pone.0020218 (PMC3112151; doi:10.1371/journal.pone.0020218)
Supplement: Table S1 — Demographic data vs. numbers of MLD patients born in Poland. (DOC) [file pone.0020218.s001.doc]

Supplemental Table 1. Demographic data vs. numbers of MLD patients born in Poland.

| Year of birth | Number of births in thousands | MLD patients born in this year | Cumulative number of MLD patients |
| --- | --- | --- | --- |
| 1960 | 669.5 | 1 | 1 |
| 1961 | 627.6 | 1 | 2 |
| 1962 | 599.5 | 0 | 2 |
| 1963 | 588.2 | 0 | 2 |
| 1964 | 562.8 | 1 | 3 |
| 1965 | 546.4 | 0 | 3 |
| 1966 | 530.3 | 1 | 4 |
| 1967 | 520.4 | 2 | 6 |
| 1968 | 524.2 | 0 | 6 |
| 1969 | 531.1 | 1 | 7 |
| 1970 | 546 | 0 | 7 |
| 1971 | 562.3 | 0 | 7 |
| 1972 | 575.7 | 0 | 7 |
| 1973 | 598.6 | 1 | 8 |
| 1974 | 621.1 | 1 | 9 |
| 1975 | 643.8 | 2 | 11 |
| 1976 | 670.1 | 2 | 13 |
| 1977 | 662.6 | 1 | 14 |
| 1978 | 666.3 | 2 | 16 |
| 1979 | 688.3 | 5 | 21 |
| 1980 | 692.8 | 1 | 22 |
| 1981 | 678.8 | 2 | 24 |
| 1982 | 702.4 | 2 | 26 |
| 1983 | 720.8 | 1 | 27 |
| 1984 | 699 | 3 | 30 |
| 1985 | 677.6 | 3 | 33 |
| 1986 | 634.7 | 2 | 35 |
| 1987 | 605.5 | 2 | 37 |
| 1988 | 587.7 | 1 | 38 |
| 1989 | 562.5 | 2 | 40 |
| 1990 | 545.8 | 0 | 40 |
| 1991 | 546 | 2 | 42 |
| 1992 | 513.6 | 2 | 44 |
| 1993 | 492.9 | 2 | 46 |
| 1994 | 481.3 | 1 | 47 |
| 1995 | 433.1 | 6 | 53 |
| 1996 | 428 | 2 | 55 |
| 1997 | 412.7 | 3 | 58 |
| 1998 | 397 | 3 | 61 |
| 1999 | 382 | 0 | 61 |
| 2000 | 378.3 | 3 | 64 |
| 2001 | 368.2 | 1 | 65 |
| 2002 | 353.8 | 1 | 66 |
| 2003 | 351 | 2 | 68 |
| 2004 | 356.1 | 3 | 71 |
| 2005 | 364.4 | 0 | 71 |
| 2006 | 374.2 | 0 | 71 |
| 2007 | 387.9 | 0 | 71 |
| 2008 | 414.5 | 1 | 72 |
| 2009 | 417.6 | 1 | 73 |
